# Dual Roadside Seismic Sensor for Moving Road Vehicle Detection and Characterization

**DOI:** 10.3390/s140202892

**Published:** 2014-02-12

**Authors:** Hua Wang, Wei Quan, Yinhai Wang, Gregory R. Miller

**Affiliations:** 1 School of Transportation Science and Engineering, Harbin Institute of Technology, No. 73 Huanghe Rd., NanGang Dist., Harbin 150090, China; E-Mail: weiquan@hit.edu.cn; 2 Department of Civil & Environmental Engineering, University of Washington, Box 352700, Seattle, WA 98195-2700, USA; E-Mails: yinhai@uw.edu (Y.W.); gmiller@uw.edu (G.R.M.)

**Keywords:** generalized cross correlation, time delay of arrival(TDOA), source localization, axle detection, vehicle detection

## Abstract

This paper presents a method for using a dual roadside seismic sensor to detect moving vehicles on roadway by installing them on a road shoulder. Seismic signals are split into fixed time intervals in recording. In each interval, the time delay of arrival (TDOA) is estimated using a generalized cross-correlation approach with phase transform (GCC-PHAT). Various kinds of vehicle characterization information, including vehicle speed, axle spacing, detection of both vehicle axles and moving direction, can also be extracted from the collected seismic signals as demonstrated in this paper. The error of both vehicle speed and axle spacing detected by this approach has been shown to be less than 20% through the field tests conducted on an urban street in Seattle. Compared to most existing sensors, this new design of dual seismic sensor is cost effective, easy to install, and effective in gathering information for various traffic management applications.

## 1. Introduction

Over the past decades, sensor manufacturers have developed various technologies for vehicle detection (see e.g., [1]). It is common to separate vehicle detection sensors into two categories based on their installation position relative to the pavement: (i) in-roadway sensors; and (ii) over-roadway sensors.

In-roadway sensors are embedded in the pavement layers or the subgrade. The main types of in-roadway sensors are the inductive loop detectors, piezoelectric sensors, magnetometers and other type of detectors. Because these sensors are installed in the traffic lanes, vehicle must pass over them in order to be detected. The installation and maintenance of such devices, therefore, requires lane or road closure, effectively stopping or impeding traffic flow. The operational conditions of in-roadway sensors can be degraded with pavement deterioration, improper installation and weather-related effects, and may be damaged by street and utility repairs. As a result, in-roadway sensor technologies require effective and careful installation, testing, and repair [2].

Over-roadway sensors are mounted either alongside or above the traffic lanes. Video detection systems, active and passive infrared, microwave radar, ultrasonic, and passive acoustic sensors belong to this category [3]. Video cameras are commonly mounted on tall poles or on traffic signal mast arms above the road. Other over-roadway sensors are installed at lower heights alongside the road. Over-roadway sensors provide a viable alternative to inductive-loop sensors. According to [1], over-roadway sensors are becoming more popular as sources of real-time data for traffic signal control and traffic management. This is because of their ability to provide multi-lane data from a single sensor, reduce maintenance costs, increase safety to installation personnel, richer data sets not available from loops or magnetometers, and competitive purchase and installation costs. When a sensor is installed directly over the lane of traffic that it is intended to monitor, its view and hence its ability to collect data are typically not obstructed. But when a sensor is mounted on the side of a roadway and views multiple lanes of traffic at a perpendicular or oblique angle to the direction of traffic flow, tall vehicles can block its view of distant lanes, potentially causing an undercount or false average speed measurement [3]. Some over-roadway sensors can be affected by weather conditions, such as wind, fog, blowing snow and rain. Another disadvantage is that installation and maintenance can require lane closure for safety purposes when it is mounted above the road.

In order to overcome the limitations of both the in-roadway and over-roadway sensors, the use of seismic signals for moving vehicle detection is proposed. In this paper, a detection configuration based on two seismic sensors installed on the road shoulder is designed. This technology may be deployed as an alternative to traditional in-roadway and over-roadway sensors. Because such sensors are installed at ground level but outside the travel lanes, installation and maintenance can be performed without diverting traffic or altering the road surface, and thus can substantially reduce costs. By recording seismic signals in each interval, we believe the time delay of arrival (TDOA) can be estimated using a generalized cross-correlation approach with phase transform (GCC-PHAT). The slope of the TDOA curve in the linear region may be used to estimate axle speed. Various kinds of vehicle characterization information, including vehicle speed, axle spacing, and driving direction, should also be extracted from the collected seismic signals. To realize these data, however, suitable algorithms must be developed to process the observed ground waves at the sensor pair, and this is the primary focus of this paper.

The remainder of this paper is organized as follows. Section 2 explains the mechanism of seismic waves caused by moving vehicles, and presents theories relevant to source localization. In particular, GCC-PHAT method is introduced to estimate the TDOA of seismic sources. Section 3 describes the basic seismic propagation model for moving vehicles that defines fundamental geometric and vehicle characteristic parameters. In Section 4, estimation methods for vehicle information, including vehicle speed, axle spacing, axle detection, and driving direction, are investigated. Section 5 reports experimental results that confirm that paired seismic sensors can be used to detect moving vehicles and estimate associated vehicle information. Finally, conclusions and future work are discussed in Section 6.

## 2. Theoretical Background

Early publications regarding automated acoustic vehicle recognition algorithms were focused mainly on military vehicle signals, in order to develop a system that improves surveillance for security [4–6]. Compared to acoustic signals, using seismic signals for detection allows the performance to be independent of wind conditions, which can often cause difficulties in acoustic detection. Moreover, seismic waves are less sensitive to factors such as acoustic noise, Doppler effects. Seismic sensors offer many benefits over acoustic and magnetic sensors, in that the propagation through the earth is less sensitive to atmospheric conditions, such as wind, moisture, and temperature [7,8]. Seismic sensors also provide non-line-of-sight detection capabilities for vehicles at significant ranges [9–11]. Seismic sensors provide good detection range, increased detection capabilities and have been extensively used in many applications [12–14]. Xin Jin *et al.* [15] presents a symbolic feature extraction method for target detection and classification, where the features are extracted as statistical patterns by symbolic dynamic modeling of the wavelet coefficients generated from time series of seismic and PIR sensors. The potential of exploiting seismic surface waves, in particular Rayleigh waves, for military vehicle and personnel tracking was investigated [16,17]. J. Huang *et al.* [18] proposed wavelet packet manifold (WPM) which provides a more robust representation for seismic target classification in Unattended ground sensor (UGS) systems. Dan Li *et al.* [19] have provided some promising preliminary results on classifying between wheeled and tracked vehicles. Outcome was positive regarding military vehicles. However, it was found their method was not suitable for non-military passenger vehicles as signals generated by military are louder and more distinguishable [4,9,20,21].

### 2.1. Vehicle Induced Seismic Waves

In the field of pavement dynamics, a vehicle can be regarded as a set of moving loads acting on the pavement, with the pavement modeled as a beam, plate or a multi-layered system on a viscoelastic foundation [22]. Within this framework, a source-path-receiver scenario can be used to characterize vehicles in terms of induced seismic signals. Vehicles' contact with irregularities on the road surface induce dynamic loads on the pavement [23]. When a vehicle, such as a car or a truck, strikes an irregularity on the road surface, it generates an impact load and an oscillating load due to the subsequent “axle hop” of the vehicle. The impact load generates seismic waves that are predominant at the natural seismic frequencies of the soil whereas the axle hop generates seismic excitation at the hop frequency. In contrast to irregularities, such as cracks, uneven manhole covers or potholes, normal road surface roughness induces continuous dynamic loads on the road. If the road surface roughness includes a harmonic component, this can lead to a periodic forcing frequency and substantial seismic excitation can be induced. This effect (which is termed the washboard effect) is familiar to car drivers traveling over dirt or gravel roads with ripples.

Vehicles moving over pavement generate a succession of impacts. These disturbances propagate away from the source as seismic waves. In general, seismic waves can be classified into two categories: body waves (shear and pressure) and surface (Rayleigh) waves [10]. Body waves travel at a higher speed through the interior of the earth and propagate in three dimensions, while surface waves travel near the surface of the earth and propagate in two dimensions. Research of surface-induced seismic waves shows that 70% of energy of the impact is distributed in the surface waves and the remaining 30% of the energy is transmitted into the earth via body waves [20]. Therefore, in this paper, we focus on the surface waves generated by the moving vehicles.

### 2.2. Source Localization Theories

Source localization is an important component of a multichannel signal processing system, which in addition to localization may include other functions such as tracking, signal separation, enhancement and noise suppression. Depending on how localization is achieved, it may be alternatively referred to as source signal strength or energy estimation, time delay of arrival (TDOA) estimation and direction of arrival (DOA) estimation in various fields [24]. For far field source scenarios, it is assumed the source is far away from the sensors such that the source's contribution has the same intensity at all sensors, and so source signal strength is not used in localization [24]. DOA can be estimated by exploiting the phase difference measured at receiving sensors and is applicable when the source emits a coherent, narrow band signal [25–29]. TDOA requires accurate measurements of the relative time delay between sensors and is suitable for broadband source localization and has been extensively investigated [30–33]. Source localization based on acoustic sensors has been used in numerous applications. In sonar signal processing, the focus is on locating underwater acoustic sources using an array of hydrophones [34]. In video conference and multimedia human computer interface applications, microphone arrays have been developed to locate and track speakers' head positions in a room environment [35]. Acoustic signatures have also been used to estimate vehicle locations in an open-field sensor network [36]. However, acoustic vehicle recognition will be affected by Doppler effects, by noises introduced from various moving parts of vehicles, and by atmospheric and terrain variations, while seismic waves are less sensitive to these factors [10].

Based on different processing domains, localization methods for seismic signals can be classified into three categories: (i) time domain methods; (ii) frequency domain methods; and (iii) time-frequency domain methods. The time domain methods include time domain cross correlation, average-magnitude-difference function methods [37], LMS-type adaptive TDE [38] and adaptive eigenvalue decomposition algorithms associated with blind channel identification [39,40]. In general, time-domain analysis is not very accurate because of the interfering noise, the complicated waveforms and the variation of the terrain [10]. Most researchers use either frequency-domain or time-frequency domain methods, which include MUSIC, ESPRIT, spatial power spectrum based approaches, maximum-likelihood methods and adaptive multichannel time delay estimation methods based on blind equalization [10,41,42], linear regression methods [43,44], and the well known generalized cross-correlation(GCC) family of methods [45].

GCC method is the most commonly used method for TDOA. In fact, GCC based on a phase transform (PHAT) is the most effective method on suppressing reverberation among a class of GCC-based methods [46]. TDOA based on PHAT was chosen for this application owing to its suitability for broadband applications, simplicity, its modest computational requirements making it suitable for real-time implementation. For the work presented here, a generalized cross-correlation approach is ideal for dual sensor-based configurations with moving sources. The details are presented in the next section.

### 2.3. Generalized Cross-Correlation Based TDOA Estimation

The problem of source localization involves the estimation of the spatial positions of signal sources from sensed data. For the case of seismic signals recorded by two sensors, this problem is usually addressed by estimating the Time Delay Of Arrival (TDOA) of a single source for the pair of sensors. We focus on TDOA estimation of a moving source for a pair of seismic sensors.

**Figure 1.**
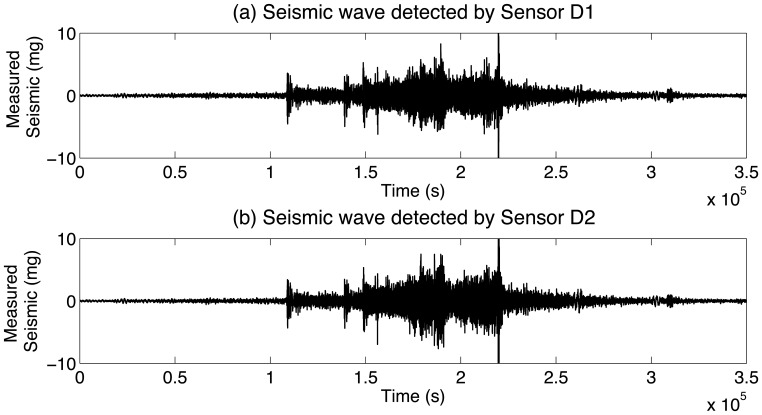
Seismic waves of vehicle detected by two sensors.

Assuming a far field source scenario, a simple TDOA estimation algorithm using paired seismic sensors can be developed based on GCC. Denoting the desired source signal by *s*[*k*], the two seismic signals *x*_1_[*k*] and *x*_2_[*k*] are expressed as:

(1) $$x_{1}[k]=s[k]+n_{1}[k]$$

(2) $$x_{2}[k]=s[k-\tau ]+n_{2}[k]$$

where *s*[*k* − *τ*] represents a delayed version of *s*[*k*], and *τ* is the time delay of the desired source. *n*_1_[*k*] and *n*_2_[*k*] represent ambient noise and more generally include interference signals in the two channels. An example of signals of the type in question can be seen in Figure 1, which shows seismic signals recorded by a pair sensors when a car passes by (the data in Figure 1 correspond to Vehicle No.1 shown in Tables 2 and 3).

**Figure 2.**
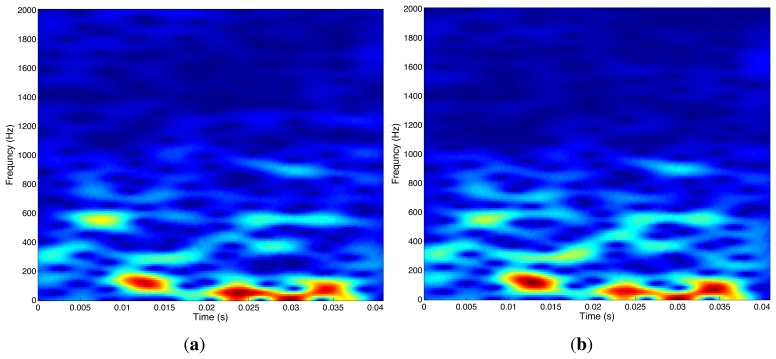
Spectrum of seismic waves detected by sensors *D*_1_ (shown in (**a**)) and *D*_2_ (shown in (**b**)).

After application of the short-time discrete Fourier transform (STFT) [47,48], *x*_1_[*k*], *x*_2_[*k*], and *s*[*k*], defined in the time domain, can be transformed into the time-frequency domain, with the transformed quantities denoted by *X*_1_(*t*, *f*), *X*_2_(*t*, *f*), and *S*(*t*, *f*), respectively. Figure 2 shows the respective time-frequency spectogram images of the two seismic signals shown in Figure 1.

The transforms *X*_1_(*t*, *f*) and *X*_2_(*t*, *f*) are given by

(3) $$X_{1}(t,f)=S(t,f)+N_{1}(t,f)$$

(4) $$X_{2}(t,f)=S(t,f)e^{-j\omega \tau }+N_{2}(t,f)$$

where *t* = 1, …, *T* and *f* = 1, …, *F* are time and frequency bin indices. It is reasonable to assume the delay time *τ* is much smaller than the time frame. *N*_1_(*t*, *f*) and *N*_2_(*t*, *f*) represent the STFT of the noise components *n*_1_[*k*] and *n*_2_[*k*], respectively. To make the above model mathematically tractable, it is assumed that the noise follows a zero-mean, frequency independent, joint Gaussian distribution.

The GCC-PHAT method [49] is among the more popular source localization methods and exhibits the best average localization performance [50]. The principle of the GCC-PHAT method consists in constructing a function *ϕ*(*τ*) of TDOA *τ* whose peak indicates the TDOA of the source. Existing methods typically extract the spatial information in a time-frequency bin (*t*, *f*) from the empirical covariance matrix *R̂_XX_*(*t*, *f*) of the input signal, which can be computed in the neighborhood of each time-frequency bin (*t*, *f*) as [51]:

(5) $${\hat{R}}_{XX}(t,f)=\frac{\sum_{t^{\prime },f^{\prime }}\omega (t^{\prime }-t,f^{\prime }-f)X(t^{\prime },f^{\prime })X{\left(t^{\prime },f^{\prime }\right)}^{H}}{\sum_{t^{\prime },f^{\prime }}\omega (t^{\prime }-t,f^{\prime }-f)}$$

where *ω* is a time-frequency windowing function of length *L_f_* × *L_t_* defining the size and the shape of the neighborhood, and (·)*^H^* denotes the Hermitian transposition operator.

By assuming that the direct seismic waves of a moving source predominates in each time-frequency bin, the TDOAs of this source *τ* are estimated from the phase difference between the two sensors represented by the argument of *R̂_XX_*(*t*, *f)*_1,2_ . The local angular spectrum is then defined as [51]:

(6) ϕGCC–PHAT(t,f,τ)=Re(R^XX(t,f)1,2|R^XX(t,f)1,2|e−2iπfτ)

where Re(*z*) denotes the real part of a complex number *z*. Equation (6) is computed in each time-frequency bin (*t*, *f*) for all discrete values of *τ* lying on a uniform grid in the range of possible TDOAs. This function is chosen so that it is likely to exhibit large values for the TDOAs of the source that are active in this time-frequency bin. In order to strengthen the estimation process and to overcome the spatial aliasing ambiguity occurring at high frequencies, the function *ϕ*^GCC–PHAT^(*t*, *f*, *τ*) is summed over all frequencies. Then, it is reduced to a single dimension to obtain the angular spectrum from which the TDOAs are estimated [50]. This is typically done by summing over all time frames [46] as:

(7) $$\phi^{\text{sum}}(\tau )=\sum_{t=1}^{T}\sum_{f=1}^{F}\phi^{\text{GCC}–\text{PHAT}}(t,f,\tau )$$

**Figure 3.**
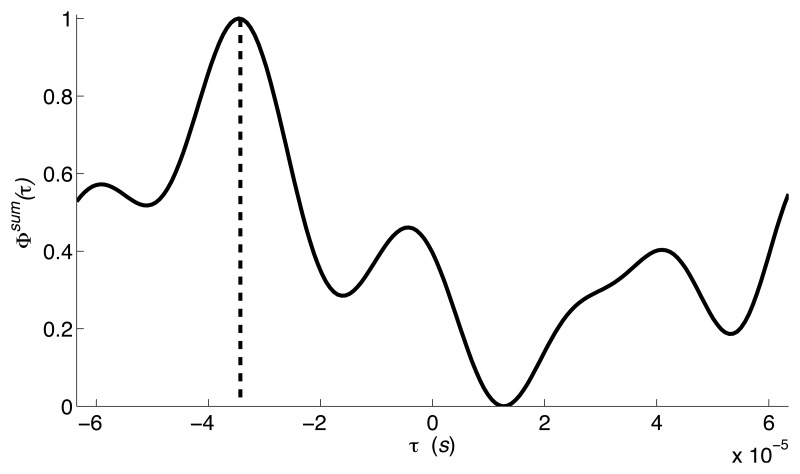
TDOA estimation for moving sources. Results correspond to captured vehicle data depicted in Figure 2.

Figure 3 shows the estimation of TDOA for the interval period shown in Figure 2. The peak shown in Figure 3 indicates that there is a seismic source whose TDOA is 3.46 × 10^−5^ s.

### 2.4. Methods of Moving Source Localization

Given the ability to estimate TDOAs for seismic signals, it is still necessary to handle moving sources. There are two main approaches to moving source localization. One approach involves formalization and prediction of the motion of the source using a dynamic (continuous time) model. The advantage of this approach is that the whole motion history can be utilized. However, such modeling can be difficult in practice [52]. In the second approach, the source is considered to be fixed over discretized time intervals and localization is performed at each interval. This approach has been widely adopted because a number of methods can be used for localization of a fixed source, such as those based on correlation functions between two sensors as described above.

## 3. Seismic Propagation Model Of Moving Vehicles

In this section the necessary framework is developed to relate TDOA estimations for moving sources to vehicle characteristics of interest for ITS purposes. This includes the quantification of the basic road/vehicle/sensor geometry and interactions, and the relation of TDOA to vehicle motion.

**Figure 4.**
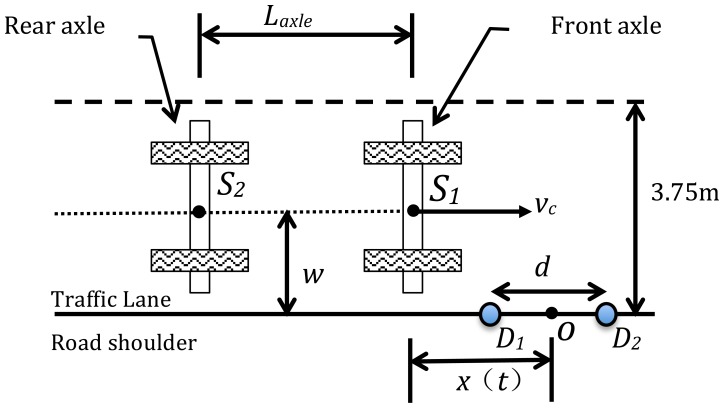
Geometry of a vehicle driving in a traffic lane.

In Section 2, seismic waves propagating in a road are generated by the dynamic loads imparted to the road structure by the wheels of the vehicles. Therefore, we consider a general scenario as shown in Figure 4, in which a vehicle with two axles is driven in a traffic lane. Two moving axles impact the pavement surface and each axle is modeled as a moving impulsive force applied on the roadway. It is reasonable to assume the two moving axles behave as two seismic sources, *S*_1_ and *S*_2_, respectively. Two seismic sensors, *D*_1_ and *D*_2_, are installed on the road shoulder, and are used to detect seismic waves propagating in the pavement. The distance between the two sensors is *d*. The width of the lane is known (3.75 m). In the case that a vehicle drives along the middle of the lane, the distance *w* from the sources (*S*_1_ and *S*_2_) to the sensors is half the lane width. The axle spacing is *L*_axle_, and *x*(*t*) is the horizontal distance between the seismic source and the center point *O* of the two sensors. Assuming the vehicle maintains a constant speed *υ_c_*, the horizontal distance can be expressed as:

(8) $$x(t)=\upsilon_{c}\cdot t$$

where *t* is the time it takes for the moving source to reach the center point *O* (*i.e.*, *t* = 0 when the moving source arrives at the center point *O* in the *x* direction).

**Figure 5.**
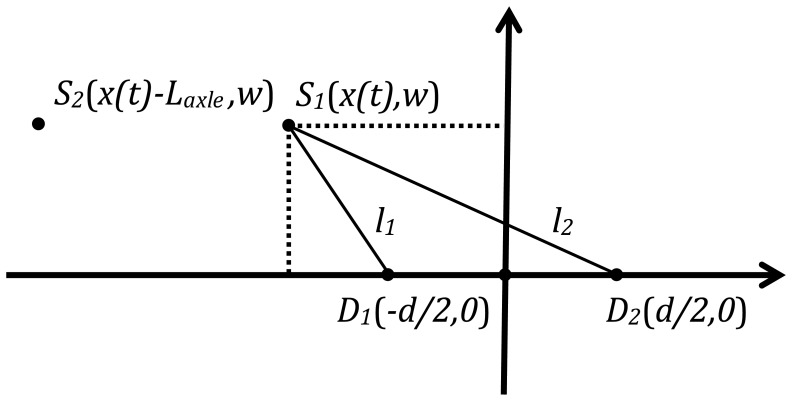
Seismic propagation model of moving vehicle.

By setting *O* as the origin of coordinates, the driving direction as the horizontal ordinate, the road plane as the coordinate plane, and noting that the velocity of a seismic wave is *υ_s_*, this scenario can be studied using the seismic propagation model of a moving vehicle as shown in Figure 5.

Here, *l*_1_ and *l*_2_ are the distances from source *S*_1_ to sensor *D*_1_ and *D*_2_, respectively. Then, the TDOA of the moving sources, *τ*(*t*), that applies to the two sensors can be determined as:

(9) $$\tau (t)=\frac{l_{1}-l_{2}}{\upsilon_{s}}=\frac{\sqrt{{\left[x(t)-(-\frac{d}{2})\right]}^{2}+w^{2}}-\sqrt{{\left[x(t)-\frac{d}{2}\right]}^{2}+w^{2}}}{\upsilon_{s}}$$

Solving this equation for *x*(*t*) leads to

(10) $$x(t)=\frac{\tau (t)\cdot \upsilon_{s}}{2}\sqrt{1+\frac{4w^{2}}{d^{2}-{\left(\tau (t)\cdot \upsilon_{s}\right)}^{2}}}$$

As the vehicle approaches the center point of the sensor pair, *τ*(*t*) → 0, which implies *d* ≫ *τ*(*t*) · *υ_s_*, and so

(11) $$\frac{4w^{2}}{d^{2}-{\left(\tau (t)\cdot \upsilon_{s}\right)}^{2}}\approx \frac{4w^{2}}{d^{2}}$$

for small *τ*(*t*). This allows the following linear approximation

(12) $$x(t)\approx \left(\frac{\upsilon_{s}}{2}\sqrt{1+\frac{4w^{2}}{d^{2}}}\right)\tau (t)$$

Because *υ_s_*, *d*, and *w* can be considered constants when a vehicle drives in a traffic lane in normal fashion, this relation can be written in the simpler form as:

(13) x(t)≈C⋅τ(t)

where

(14) $$C=\frac{\upsilon_{s}}{2}\sqrt{1+\frac{4w^{2}}{d^{2}}}$$

When a moving vehicle runs along a traffic lane, the vehicle speed is equal to the rate of change of *x*(*t*), that is, *υ_c_* = *dx*/*dt*. Using Equation (13), we can write

(15) $$\upsilon_{c}=C\frac{d\tau (t)}{dt}$$

Equation (15) shows that the vehicle speed is given by a linear relationship with respect to the derivative of *τ*(*t*) in the case *τ* → 0. Thus, the speed of the vehicle can be estimated from TDOA data using Equation (15).

## 4. Estimation of Moving Vehicle Information

Generally, vehicle speed, axle spacing, detection of both vehicle axles and moving direction are major parameters associated with the characterization of moving vehicles in traffic lanes. This section presents techniques for quantifying these parameters from roadside seismic sensor data using the framework and relations presented in the previous section.

### 4.1. Vehicle Speed Estimation

**Figure 6.**
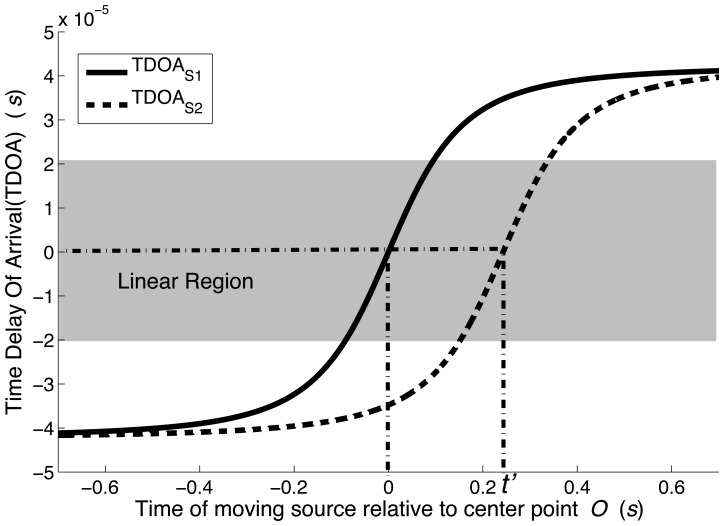
Analytical curve of TDOA *vs.* relative time for moving vehicle at 40 km/h.

Equation (15) shows that the vehicle speed *υ_c_* is proportional to the slope of *τ*(*t*) evaluated at *τ* → 0. However, *τ*(*t*) can not be set extremely small for the limitation of time interval. Here, the slope of *τ*(*t*) is estimated in the region which *τ*(*t*) is from −2 × 10^−5^ s to 2 × 10^−5^ s, which is considered as the linear region. Figure 6 shows a plot of TDOA *τ_i_*(*t*) from two sources plotted relative to the time of the moving source *t*. Time *t* = 0 corresponds to the vehicle reaching point *O*. The curves shown in Figure 6 were obtained using Equation (9) assuming a vehicle speed of 40 km/h. The solid line TDOA*_S_*_1_ represents the moving source *S*_1_, which is the front axle shown in Figure 4. In the region near *τ*(*t*) = 0, shown as the shaded region in Figure 6, the TDOA*_S_*_1_ curve shows a proportional relationship as expressed in Equation (15). Outside this region, the relationship becomes non-linear, as expected.

**Figure 7.**
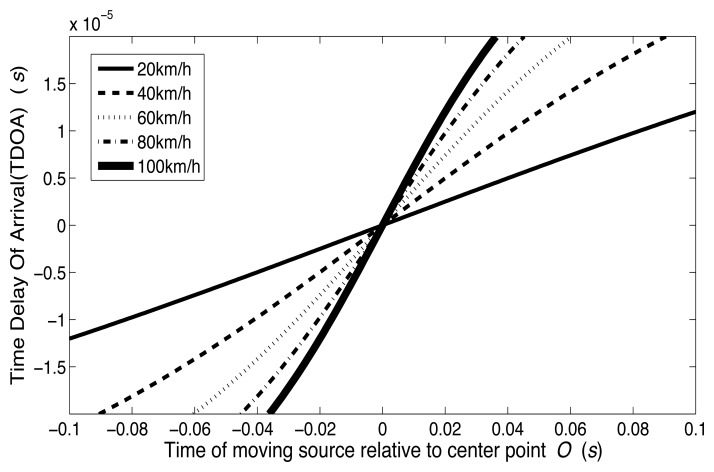
Analytical curve of TDOA *vs.* relative time for vehicle speeds of 20 km/h, 40 km/h, 60 km/h, 80 km/h, and 100 km/h, respectively.

For the linearly varying *τ*(*t*) in the shaded portion of Figure 6, a general linear equation can be introduced as:

(16) y=m⋅(x+Δt)

where *m* is the slope of the curve and Δ*t* is a time offset. The linear least squares fitting technique is the simplest and most commonly applied form of linear regression and provides a solution to the problem of finding the best fitting straight line through a set of points. This technique is applied to estimate the curve slope *m* [53]. Figure 7 shows five curves corresponding to 20 km/h, 40 km/h, 60 km/h, 80 km/h and 100 km/h in the approximately linear region. The figure shows that as speeds increase, the slopes of the curves also increase as expected. The linear least squares fitting technique can be applied to calculate the slope of the five curves shown in Figure 7. The estimated speeds obtained from Equation (15) are shown in Table 1. The smallest estimated error occurs at low vehicle speed (see 20 km/h), and the largest estimated error of −2.76% at the highest speed (see 100 km/h). It can be seen from Equation (11) that the estimated error is caused by the approximation associated with assuming *τ*(*t*) → 0.

In an actual application, the speeds of the front axle, rear axle, and any additional axles are estimated when a vehicle with two or more axles passes by the seismic sensors. This provides an oversampling mechanism to reduce the effects of noise on any individual signal, and so the estimated speed of a vehicle can be based on the average value of the speed calculated from all the axles.

### 4.2. Estimation of Axle Spacing

The dashed line in Figure 6 is TDOA*_S_*_2_, the TDOA curve of the moving source *S*_2_ corresponding to the rear axle shown in Figure 4. This curve has a time shift relative to line TDOA*_S_*_1_. The time shift is associated with the axle spacing between the front and rear axles. As shown in Figure 5, the rear axle (source *S*_2_) is offset from the front axle by the time required to traverse the axle spacing *L*_axle_ at the vehicle speed *υ_s_*.

In the procedure of slope estimation shown as Equation (16), the time offset Δ*t* of two sources *S*_1_ and *S*_2_ also could be estimated and set to Δ*t*_1_ and Δ*t*_2_, respectively. Then, the time shift *t′* between sources *S*_1_ and *S*_2_ is obtained by:

(17) $$t^{\prime }=|\Delta t_{1}-\Delta t_{2}|$$

Therefore, the axle spacing *L*_axle_ can be obtained from

(18) $$l_{\text{axle}}=\upsilon_{c}\cdot t^{\prime }$$

### 4.3. Detection of Vehicle Axles and Moving Direction

As shown in Figure 4, the TDOA of two moving sources is equal to 0 as the moving axles pass by the origin of coordinates *O*. It is straightforward in principle to detect an axle by determining when *τ*(*t*) = 0. However, this method can be influenced strongly by noise in the system. Instead of using *τ*(*t*) = 0 for axle detection, the time offset Δ*t* is applied to indicate that there is an axle passing the origin of coordinates *O*.

As for the direction of motion of passing vehicles, the sign of the curve slope *m* indicates the direction. If *m* > 0, then the vehicle is driving from sensor *D*_1_ to sensor *D*_2_. Conversely, *m* < 0 indicates that the vehicle moving from sensor *D*_2_ to sensor *D*_1_.

## 5. Experimental Field Studies

To investigate the ability of roadside seismic sensors to detect and quantify basic vehicle characteristics, a field study was carried out in Seattle. Two seismic sensors were installed on the shoulder of a two-lane road, which is located at Northeast Stevens Way in Seattle. This road is rigid (concrete) pavement and was rebuilt three years ago. We chose this road as the experimental field because traffic conditions and pavement conditions represented the average conditions of an urban road in Seattle.

### 5.1. Experimental Setup

The sensors were located on the road shoulder and close to the traffic lane. The two sensors were attached to the road shoulder by use of adhesive which gives an excellent mounting method to provide reliable results for the experiments. The model of the two sensors was 393B12. The sensor sensitivity was 10,000 mV/g. An amplifier with a 100×-magnification factor was used and its bandwidth extended from 0 Hz to 10 kHz. A data acquisition device was used to record seismic data with 16-bit resolution and a 100-kHz sampling rate. The measuring range was from −10 mg to +10 mg. For locating moving vehicles, the recorded data were split into 40.96 ms time intervals (*i.e.*, 4,096 sampling points in each interval in the case of 100 kHz sampling rate). In each time interval, TDOA was calculated by the GCC-PHAT approach presented above. In order to achieve higher resolution in frequency, the STFT was computed with half-overlapping sine windows of length 4,096 since this gave the best results in our preliminary experiments. The time-frequency windowing function *ω* in Equation (5) was a Hanning window. Its size was set to *L_f_* = 1, 024 and *L_t_* = 1,024 for all angular spectrum-based methods since this gave the best results in our preliminary experiments. The seismic sensor spacing was *d* = 0.127 m, so spatial aliasing occurs above 7.5 k Hz. The distance from the sensors to the middle of the traffic lane was *w* = 1.9 m. The seismic wave velocity was *υ_s_* = 1, 900 m/s for the concrete material [54].

The seismic signals induced by 18 representative vehicles was recorded. A video camera was set up nearby the experimental apparatus to record the vehicles. There were 16 vehicles had two axles. They were 7 cars, 4 SUVs, 4 trucks and 1 motorbike, in total. Another 2 vehicles were bus with three axles. By analyzing the video recording, vehicle speed, axle number and axle spacing of those 18 vehicles were measured. The video-based ground-truth data are shown in Tables 1, 2 and 3, respectively.

### 5.2. Experimental Results and Discussion

**Figure 8.**
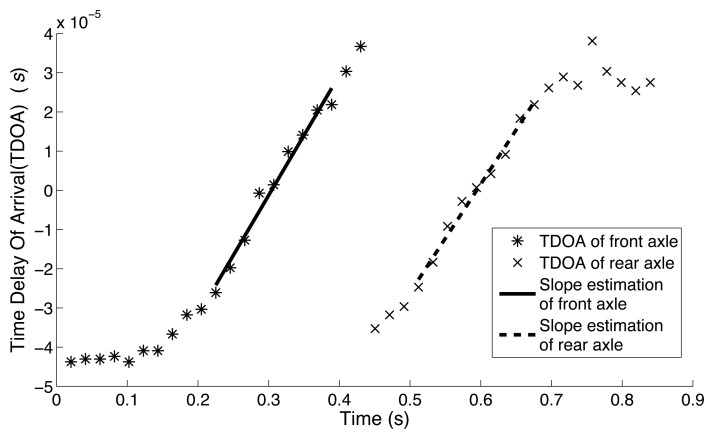
Time delay of arrival (TDOA) as Vehicle No.1 passes by.

Figure 8 shows TDOA curves when Vehicle No.1 (in Table 2) passed by the sensor pair. The seismic signals in the time domain were shown earlier in Figure 1. For obtaining the slope of TDOA, the linear least squares fitting technique was applied. The intervals over which TDOA is less than 2 × 10^−5^ s were used to fit the line for the linear region. Both the solid line and dash line in Figure 8 are fitted lines and represent the front axle and rear axle of Vehicle No.1, respectively.

As shown in Table 2, the estimated speeds of the front and rear axles of Vehicle No.1 are 43.75 km/h and 45.34 km/h, respectively. The estimated speed of this vehicle is the average value of the two axle speed results and is 44.55 km/h. By use of video analysis, the actual vehicle speed was determined to be 40.28 km/h. The estimation error in this case is thus 4.27 km/h or 10.6%. Table 2 also shows estimated results for the other 17 vehicles. The results indicate that the estimated speed error is less than 20%.

**Figure 9.**
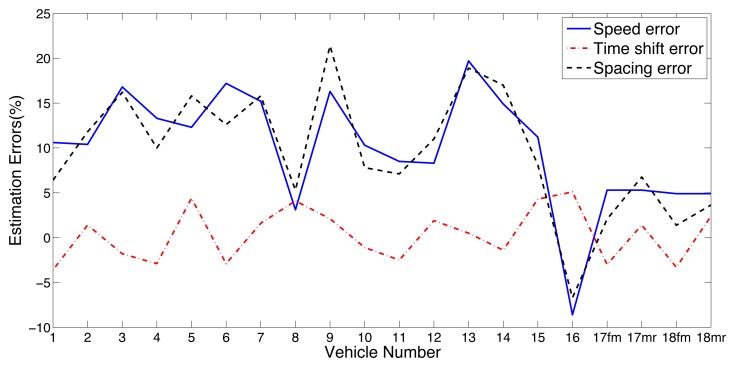
Error curves of speed, axle spacing and time shift.

Table 3 shows the estimation of time shift of each vehicle. The time shift is calculated based on the method discussed in Section 4.3. The estimated axle spacings for 18 vehicles were obtained by Equation (18) based on the results shown in Tables 2 and 3. The results show that the estimation error for axle spacing is around 20%. The error curves of speed, axle spacing and time shift are shown in Figure 9.

The detection of vehicle axles and moving direction based on the methods introduced in Section 4.3 were applied to the data. The axles of all vehicles were detected reliably, with a rate of axle detection of 100%. The detection of the vehicle direction was also carried out. The driving direction of all 18 vehicles is from Sensor *D*_1_ to Sensor *D*_2_. The detection of vehicle direction was also 100% accurate.

Most of the speed errors shown in Figure 9 are positive. It means that the vehicle speeds are constantly overestimated. By analyzing the recorded video, it was found that the vehicles were driven close to shoulder instead of down the middle of the lane. In Section 3, we assumed that vehicles drive along the middle of the lane and the distance *w* is also considered as a constant value. This caused the actual distance *w* is less than assumed one. Based on Equations (14) and (15), the estimated speed of vehicles was greater than the actual speed in this case. In other words, estimated speed is more sensitivity to distance *w*.

As can be seen from Figure 9, the error of time shift is less than the other two error results and shows random distribution. For the axle spacing is equal to vehicle speed multiplied by the time shift. The error of vehicle speed was propagated to axle spacing estimation. Therefore, the speed error influences the results of axle spacing estimation as shown in Figure 9.

## 6. Conclusion

This paper presented a dual seismic sensor approach for detecting and characterizing moving vehicles with respect to vehicle speed, axle spacing and axle detection, and direction of vehicle travel. The two seismic sensors were installed on the road shoulder rather than in the road or over the road. This roadside dual seismic sensor configuration is designed to overcome the installation, maintenance, and operational limitations of existing in-roadway and over-roadway sensors.

In this paper, a seismic signal propagation model of a moving vehicle as a function of source-to-sensor distance was first established. Based on this model, the seismic source localization problem was formulated as a generalized cross-correlation with phase transform, denoted as GCC-PHAT. TDOA estimation based on only two sensors was considered. The slope of the TDOA curve in the linear region is applied to estimate axle speed. Based on this linearization, estimation methods for vehicle speed, axle spacing and detection, and driving direction were presented.

Field measurements in actual traffic situations were carried out in order to test the effectiveness of the approach. The results obtained from the experiments have been compared to the ground truth. It was demonstrated that the proposed approach measured speed within an estimated error of about 20%.

Future work includes improvement of the estimated accuracy of vehicle information, and hardware implementation of the dual seismic sensor approach. Moreover, we should demonstrate the robustness of the applied model in different weather conditions (e.g., rainy or windy conditions). And for general purpose, proposed approach will be investigated in both asphalt and concrete pavement. Also, the ability to detect additional individual vehicle characteristics should be investigated as multiple vehicles will be introduced in the next steps. Meanwhile, other ground-truth measurement methods should be investigate to improve their accuracy beyond the capabilities of video measurement methods.

## Figures and Tables

**Table 1.** Speed estimation in linear region of TDOA.

**Vehicle Speed (km/h)**	20	40	60	80	100
**Estimated Speed (km/h)**	19.97	39.82	59.38	78.56	97.24
**Error (%)**	−0.15	−0.45	−1.03	−1.8	−2.76

**Table 2.** Results of speed estimations of 18 vehicles.

**Vehicle** **No.**	**Vehicle** **type**	**Axle** **number**	**Front** **Axle Speed** **(km/h)**	**Middle** **Axle Speed** **(km/h)**	**Rear** **Axle Speed** **(km/h)**	**Est.** **Speed** **(km/h)**	**Actual** **Speed** **(km/h)**	**Speed** **Error** **(%)**
1	Car	2	43.75		45.34	44.55	40.28	10.6
2	Car	2	30.10		33.43	31.77	28.77	10.4
3	Car	2	26.79		28.57	27.68	23.70	16.8
4	Car	2	29.19		33.75	31.47	27.78	13.3
5	Car	2	28.39		28.16	28.28	25.18	12.3
6	Car	2	31.52		35.94	33.73	28.77	17.2
7	Car	2	28.06		29.95	29.01	25.18	15.2
8	SUV	2	19.72		24.00	21.86	21.20	3.1
9	SUV	2	36.15		35.94	36.05	30.99	16.3
10	SUV	2	31.40		36.94	34.17	30.99	10.3
11	SUV	2	33.46		36.49	34.98	32.23	8.5
12	Truck	2	20.73		24.03	22.38	20.66	8.3
13	Truck	2	32.43		36.42	34.43	28.77	19.7
14	Truck	2	32.11		34.02	33.07	28.77	14.9
15	Truck	2	42.83		46.75	44.79	40.28	11.2
16	Motorbike	2	41.53		40.31	40.92	44.76	−8.6
17	Bus	3	31.24	31.68	31. 37	31.43	29.84	5.3
18	Bus	3	28.14	27.93	28.46	28.18	26.86	4.9

**Table 3.** Results of both time shift of axle and axle spacing estimations.

**Vehicle** **No.**	**Actual Time** **Shift (s)**	**Est. Time** **Shift (s)**	**Time Shift** **Error (%)**	**Actual Axle** **Length (m)**	**Est. Axle** **Length (m)**	**Spacing** **Error (%)**
1	0.250	0.241	−3.6	2.8	2.98	6.4
2	0.350	0.355	1.4	2.8	3.13	11.8
3	0.400	0.393	−1.8	2.6	3.02	16.2
4	0.350	0.340	−2.9	2.7	2.97	10.0
5	0.367	0.383	4.4	2.6	3.01	15.8
6	0.383	0.372	−2.9	3.1	3.49	12.6
7	0.367	0.373	1.6	2.6	3.01	15.8
8	0.467	0.486	4.1	2.8	2.95	5.3
9	0.333	0.340	2.1	2.8	3.40	21.4
10	0.367	0.363	−1.1	3.2	3.45	7.8
11	0.317	0.309	−2.5	2.8	3.00	7.1
12	0.683	0.696	1.9	3.9	4.33	11.0
13	0.433	0.435	0.5	3.5	4.16	18.9
14	0.517	0.510	−1.4	4.0	4.68	17.0
15	0.300	0.313	4.3	3.6	3.89	8.1
16	0.117	0.123	5.1	1.5	1.40	−6.7
17_fm_*	0.699	0.678	−3.0	5.8	5.92	2.07
17_mr_**	0.929	0.942	1.4	7.7	8.22	6.75
18_fm_*	0.777	0.751	−3.3	5.8	5.88	1.38
18_mr_**	0.995	1.019	2.4	7.7	7.98	3.64

* Axle spacing between front axle and middle axle;

** Axle spacing between middle axle and rear axle.
